# GAS-STING signaling plays an essential pathogenetic role in Doxorubicin-Induced Cardiotoxicity

**DOI:** 10.1186/s40360-022-00631-0

**Published:** 2023-03-24

**Authors:** Zilong Xiao, Ziqing Yu, Chaofeng Chen, Ruizhen Chen, Yangang Su

**Affiliations:** Department of Cardiology, Shanghai Institute of Cardiovascular Diseases, Zhongshan Hospital of Fudan University, Shanghai, China

**Keywords:** Doxorubicin-induced cardiomyopathy, STING, Apoptosis, Inflammation

## Abstract

**Background:**

The severe unfavorable effects of doxorubicin on the heart restrict its clinical usage. Numerous investigations document that cyclic GMP-AMP synthase (cGAS) activator of interferon genes (STING) cascade influences inflammation along with the immune response in a variety of diseases. The pathophysiological function of the cGAS-STING cascade in Doxorubicin-induced cardiomyopathy (DIC) is, nevertheless, unknown.

**Methods:**

In vivo, cardiotoxicity was triggered by a single dose of intra-peritoneal inoculation of doxorubicin (15 mg/kg) in wild-type C57BL/6J mice and STING knockdown animals. Adeno-associated virus 9 (AAV9) was utilized to silence STING. qPCR along with Western blotting were adopted to assess alterations in the cGAS/STING cascade. To assess cardiac function, we employed echocardiography coupled with histology, as well as molecular phenotyping. In vitro, HL-1 cardiomyocytes were introduced as test models.

**Results:**

In wild type mice, doxorubicin stimulation significantly activated the cGAS/STING pathway. STING silencing increased rate of survival along with heart function in mice, as well as diminished myocardial inflammatory cytokines along with apoptosis. These observations were also confirmed by utilizing siRNA of STING in vitro studies.

**Conclusion:**

This research premise established that STING inhibition could alleviate Dox-triggered cardiotoxicity in mice. As a result, preventing DIC by repressing STING in cardiomyocytes might be a possible treatment approach.

**Supplementary Information:**

The online version contains supplementary material available at 10.1186/s40360-022-00631-0.

## Introduction

Anthracyclines are a kind of chemotherapeutic drug employed to treat a variety of solid along with haematological cancers. Nonetheless, a high cumulative dose of anthracyclines causes DIC (DOX-induced cardiomyopathy), which is a progressive, chronic, as well as life-threatening cardiomyopathy [1]. Due to its cardiotoxicity, DIC remarkably diminishes the availability of DOX for tumors, which is linked to a poorer prognosis. As a result, overcoming DOX-induced cardiotoxicity is critical for cancer patients to have a positive treatment outcome. Several pharmacological, as well as non-pharmacological approaches aimed at reducing cardiac toxicity have exhibited positive results in animal along with cell models [2]. But to date, dexrazoxane is the only drug clinically approved for the prevention of anthracycline cardiotoxicity. Thus, it is highly important to clarify the mechanisms of DOX-induced cardiotoxicity for developing more treatment strategies.

Inflammation in DIC is not triggered by infectious pathogens, however by the stimulation of endogenous substances. The cyclic GMP-AMP (cGAMP) synthase (cGAS) stimulator of interferon genes (STING) cascade is one mechanism to generate an innate immune response. Pattern-recognition receptors (PRRs) detect pathogen-linked molecular patterns, also known as danger-associated molecular patterns (DAMPs), and activate signaling cascades that culminate in the production of pro-inflammatory cytokines and type I interferons (IFN-a and IFN-b) [3]. The endoplasmic reticulum-based stimulator of interferon gene (STING), which has five putative transmembrane domains, might activate the nuclear factor (NF)- κB along with the interferon regulatory factor 3 (IRF3) transcription cascades to increase the amount of type I interferon [4]. STING has pivotal proinflammatory, as well as immunomodulatory influences in a variety of diseases, from heart disease to liver disease. Increased intestinal permeability caused by ethanol leads to the buildup of lipopolysaccharide (LPS) in liver tissue and increased endoplasmic reticulum stress in hepatocytes in alcoholic liver disease. The stress will also cause the separation of STING and the endoplasmic reticulum, which will activate IRF3 and cause hepatocyte death. However, to our knowledge, whether the cGAS/STING cascade participate in the onset of doxorubicin-induced cardiotoxicity remains unknown. As a result, the goal of this research was to establish the role the cGAS/STING cascade in DOX-triggered heart dysfunction and damage.

## Materials and methods

### Experimental animals and DOX treatment

All animal experiments performed on live animals were approved by Fudan University’s Committee on the Ethics of Animal Experiments and adhered to relevant guidelines including the ARRIVE guidelines for animal experiments in the study. Adult male C57BL/6 mice (8–10 weeks old; 23–26 g; Department of Laboratory Animal Science, Fudan University, Shanghai, China) were housed in pathogen-free settings with unrestricted access to water along with food under 12/12 h dark/light cycle. All animal experiments were granted approval by Fudan University’s Committee on the Ethics of Animal Experiments and carried out in strict conformity with the China Council on Animal Management’s requirements. Acute DOX cardiotoxicity was generated by inoculation of a single dosage (15 mg/kg) of DOX (Sigma-Aldric) intraperitoneally, as described in earlier research [5].

### rAAV9 vector production and administration

The rAAV9 vectors used here were produced and quality controlled as specified. The murine shRNA-STING (RefSep, NM 001289591.1) coded AGAGGTCACCGCTCCAAATAT were synthetized, cloned into pAAV-U6-GFP vector, as well as commercially provided by Zorin Biosciences (Shanghai, China).Viral titers for the produced vectors were as follows: 1.59 × 10^14^GC/mL for the rAAV9-sh-STING vector; 7.24 × 10^13^ GC/mL for the rAAV9-control vector. Male WT C57BL/6J mice were tail vein-injected with 3.5 × 10^11^ viral genome particles of rAAV9 in 100 µl PBS per mouse. In vivo, rAAV9-mediated gene transfer of sh-STING and control was allowed for 3 weeks. Then, mice were treated with dox as described above.

### Echocardiographic assessment

We anesthetized the mice with 1–2% isoflurane and shaved their chest hair via a hair-removing cream before undergoing transthoracic echocardiography. On the fifth day following inoculation with DOX and normal saline, an animal-distinct device (Vevo707B, Visual Sonics Inc) was utilized to execute an echocardiography. The LVEDD (left ventricular end-diastolic dimension), LVESD (left ventricular end-systolic dimension), LVEF (left ventricular ejection fraction), along with the LVFS (left ventricular fractional shortening) were assessed as previously documented after imaging of the B-and M-modes [6]. Three skilled technicians were blinded to the animal groups and conducted all assessments.

### Histopathological analysis

The cardiac tissue was immediately deposited in 10% neutral buffered formalin for 24 h after the echocardiogram. The specimen was then immersed in paraffin and serial slices of 4 _m thickness were cut. Hematoxylin and eosin (H&E) staining was performed for the evaluation of cytoplasmic.

Vacuolization and myofibrillar loss. Four section were examined, which were graded from 1 to 5, depending on the presence/absence of either cytoplasmic vacuolization or myofibrillar loss. Masson’s trichrome staining was performed to visualize fibrosis. Images of four fields of Masson’s trichrome stained slides were acquired. The percentage of fibrosis in the images having greater blue than red intensities was calculated as the staining intensity per square millimeter of area using the Image J software. TUNEL staining was utilized to reveal in situ DNA fragmentation with the DAB (SA-HRP) Tunnel Cell Apoptosis Detection Kit (Servicebio).

### Plasma biochemical analysis

Each group’s blood samples were span at 3,000°g for 15 min (4 °C) in heparinized tubes. Following that, the plasma samples were kept at -80 °C for further analysis. Plasm cardiac troponin T (cTnT) and lactate dehydrogenase enzyme (LDH) levels were measured by ELISA kits provided by Abcam to evaluate the myocardial injury. An automatic biochemical Platform (ADVIA® 2400, Siemens Ltd., China) was employed to assess the content of creatine kinase isoenzymes (CK-MB) in the blood.

### Cell culture and treatment

The American Type Culture Collection provided the mouse atrial cardiomyocytes (HL-1 cells) (ATCC). HL-1 cells were grown in DMEM media enriched with 10% FBS, 1% penicillin, along with 1% streptomycin antibiotics. At 37 degrees Celsius, cells were incubated in a 5% CO2 environment. DOX (1µmol/ml) was introduced to the media after the cells achieved 75% confluence to create a DOX-triggered cardiomyocyte damage model in vitro. Cardiomyocytes sown in 6-well plates were extracted for protein assessment along with flow cytometric analysis, in 12-well plates for RNA analysis, in 24-well plates for immunofluorescence staining analysis, and in 96-well plates for CCK8 measurement after being exposed to DOX for 24 h. Each experiment in our study was performed at least thrice, and samples in one experiment exhibited an independent replication.

### RNA interference

A siRNA was adopted to dampen STING expression. The results were compared to a control siRNA from the same provider. Lipofectamine3000 transfection reagent was adopted to deliver siRNA to HL-1 cells (Thermo Fisher Scientific). Cells were stimulated as directed after transfection (48 h).

### Cell viability

CCK8 test was utilized to assess cell viability. Cardiomyocytes were pre-inoculated (2000 cells/pore) on a 96-pore plate, then 10 µL CCK8 solution (Beyotime, Shanghai, China, C0037) was introduced to every pore. The cardiomyocyte was identified using an enzyme-labeled equipment (Thermo, America) at a wavelength of 450 nm after one hour incubation in a cell incubator.

### RNA extraction and quantitative real-time PCR

Isolationn of total mRNA was done with the Trizol Reagent (Invitrogen, USA), then cDNA was generated from 500 ng with the Takara Bio’s PrimeScript RT Master Mix, as directed by the manufacturer. The cDNA was employed as a template for qPCR, which was carried out on the StepOnePlus Real-Time PCR Platfom with the Fast SYBR Green Master Mix (Applied Biosystems). The internal control for the reactions was glyceraldehyde 3-phosphate dehydrogenase. The comparative cycle threshold (CT; 2 DDCt) approach was used to calculate relative gene expression levels.

### Western blot analyses

The hearts were instantly extracted and liquid nitrogen-frozen. RIPA lysis buffer (Beyotime Biotechnology, Nanjing, China) along with a cocktail of complete protease inhibitor were employed to isolate total protein from homogenized heart tissues. Following that, fractionation of 50 _g of extracted proteins was done on the 10% or 12% polyacrylamide gel and blotted onto PVDF membrane. Antibodies against cGAS (Cell Signaling Technology), STING (Cell Signaling Technology), TBK1 (Cell Signaling Technology), Phospho-TBK1 (Cell Signaling Technology), IRF3 (Cell Signaling Technology), Phospho-IRF3 (Cell Signaling Technology), NF-κB p65(Cell Signaling Technology), Phospho-NF-κB p65 (Cell Signaling Technology), Caspase-3 (Protein (Abcam) were adopted to assess protein expression via immunoblotting. The blots were then inoculated with radish peroxidase-linked rabbit secondary antibody immunoglobulin G (Cell Signaling Technology) after three washes. The loading control was GAPDH(Proteintech). Chemiluminescence was adopted to assess the protein bands, and a Bio-Rad with image software basic Quantity One was used to quantify them (Bio-Rad, Hercules, CA, USA).

### Flow cytometric detection of apoptosis

The Annexin V-FITC Apoptosis Cardiomyocyte apoptosis was quantified by annexin V/PI apoptosis assay (BD, CA, USA). Harvested cells were re-suspended in binding buffer after being washed with cold PBS. In 100 µl of cell suspension, annexin V and PI were introduced. After a 15-minute incubation period at room temperature in the dark, 400 µl of binding buffer was introduced to each tube. The assessment was done with a fluorescence-activated cell sorting equipment with FlowJo software.

### Statistical analysis

Data analyses were implemented in the Graph Pad Prism 9 software. Data are given as the mean or standard deviation and comparison was done with one-way analysis of variance (ANOVA) and then the Student-Newman-Keuls (SNK) post hoc test. The Kaplan-Meier estimator was adopted to create survival curves, and then comparison was done with the log-rank test. Statistical significance was shown by a *p* < 0.05.

## Results

### The expression levels of cGAS and STING in the myocardium of DIC mice

**Fig. 1:**
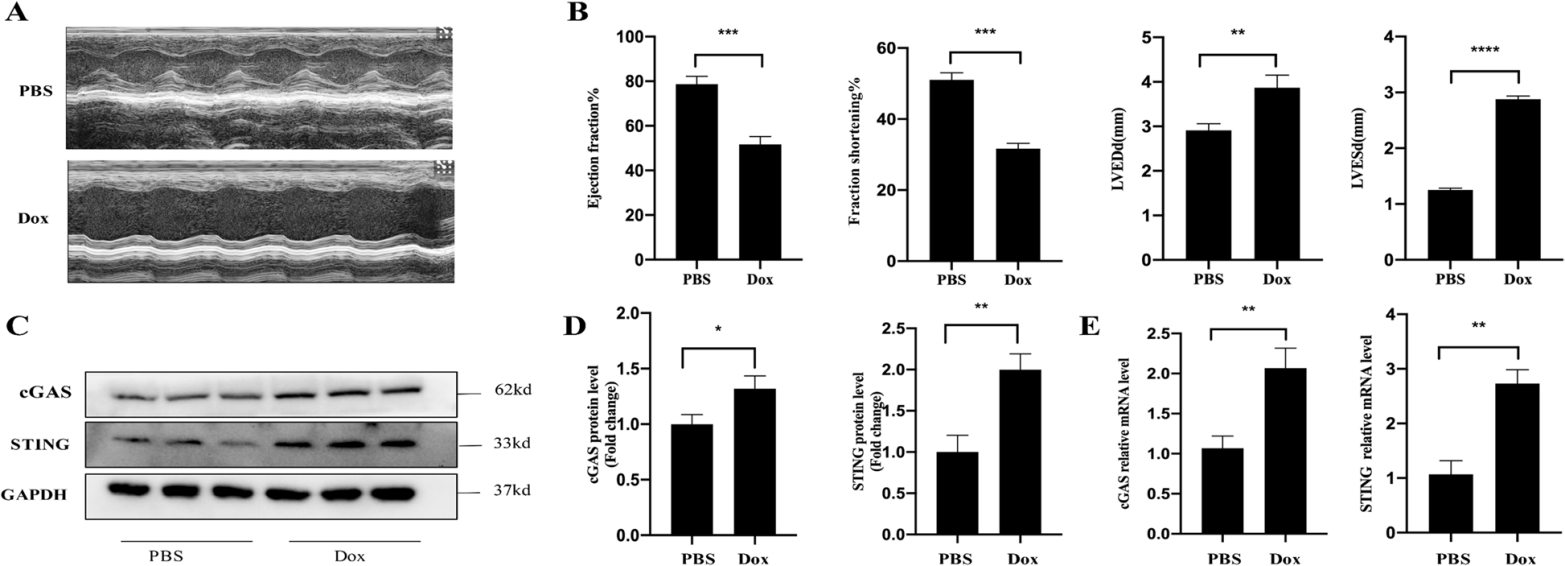
The expression levels of cGAS and STING in the myocardium of DIC mice (**A**–**E**). **A** Echocardiographic images of control mice or DIC mice at day14. **B** left ventricle ejection fraction, left ventricle fractional shortening, left ventricular end-diastolic dimension, left ventricular end-systolic dimension at day 14 (*n* = 4). **C**-**E** 15 mg/Kg doxorubicin injection enhanced cGAS and STING expression in DIC mice (day 14) by immunoblot and mRNA analysis

To characterize acute DIC in mice, we used a single dose of doxorubicin (15 mg/kg; ip) to induce DIC in mice. Body weight loss, substantial cardiac atrophy, as measured by the reduced heart weight to tibia length ratio, decreased LV ejection fraction (LVEF), and fractional shortening (FS) were all found 14 days after injection in Dox-treated rats (Fig. 1A-B). We evaluated cGAS and STING mRNA and protein expression in normal and DIC cardiac tissues on day 15. The expression of cGAS and STING was shown to be considerably higher in the hearts of mice with DIC (Fig. 1C-E).

### Knockdown of STING improved survival and cardiac function of DOX- treated mice

**Fig. 2:**
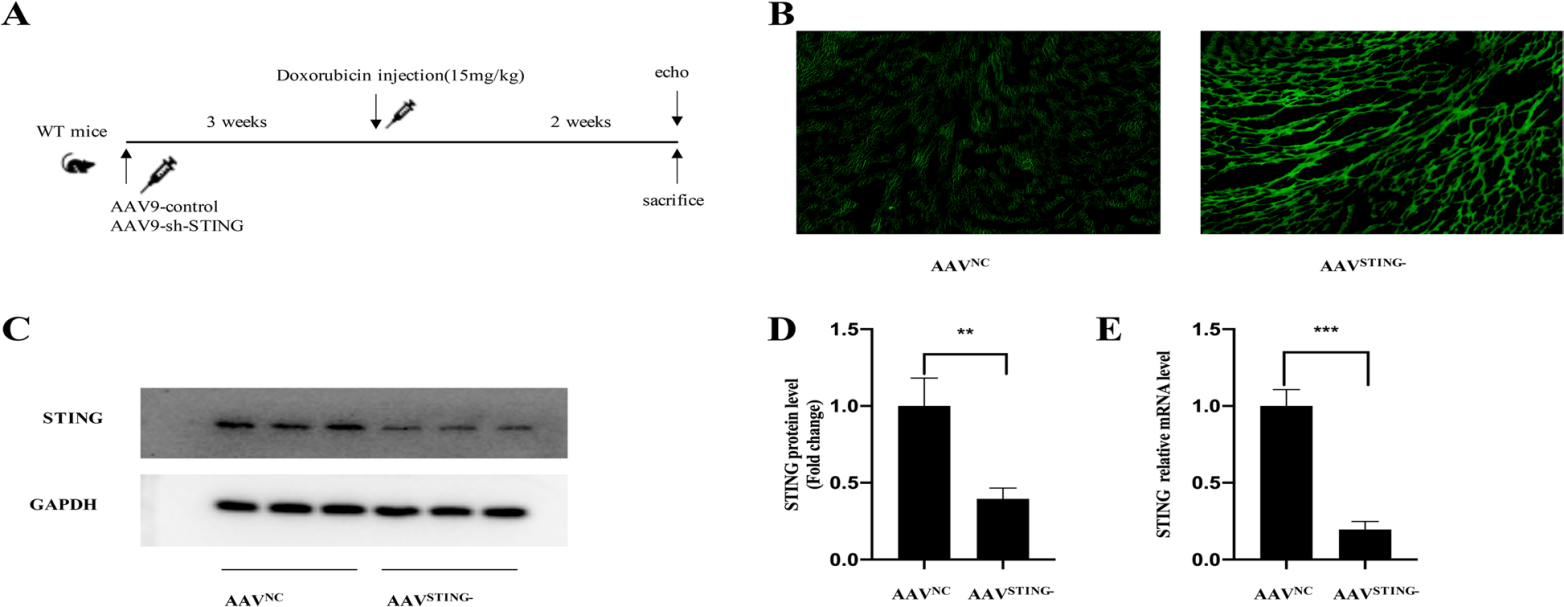
AAV9-sh-STING injection decreased heart STING expression levels of baseline conditions in vivo (**A**-**E**). **A** schematic representation of the experimental design. **B** representative images of fluorescence microscope of frozen heart left ventricular sections after 3 weeks of AAV9-control and AAV9-sh-STING injection (scale bar, 50 m). **C**-**E** immunoblot and mRNA analysis reveals AAV9-sh-STING injection reduced STING expression compared with AAV9-control injection after 3 week of injection

**Fig. 3:**
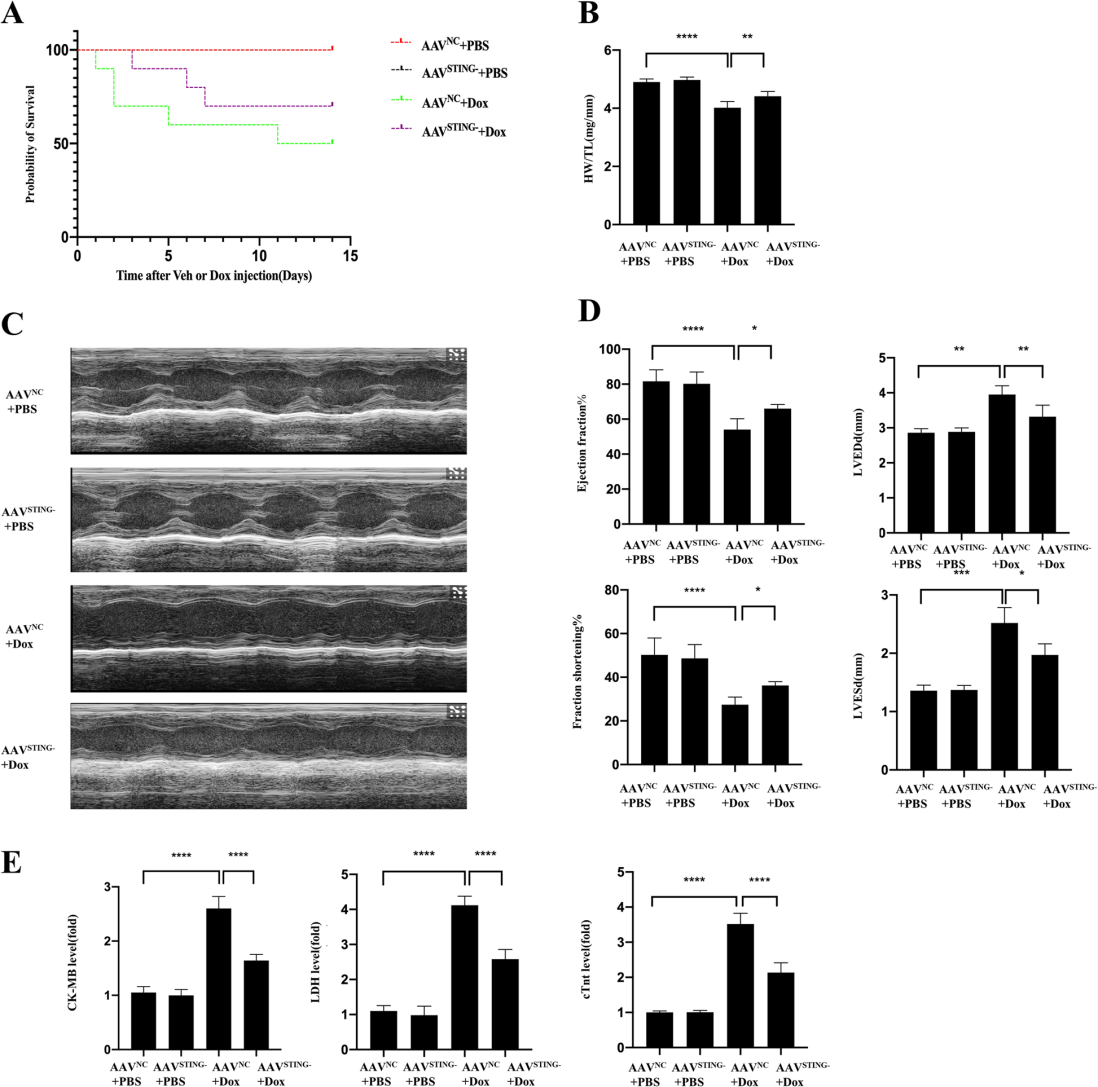
Knockdown of STING improved survival and cardiac function of DOX-treated mice (**A**-**E**). **A** Effect of STING knockdown on the 14-day survival rate after DOX injection (*n* = 10). **B** Effect of STING knockdown on HW/TW. **C** Representative echocardiographic images of each group (*n* = 4). **D** Effect of STING knockdown on left ventricle ejection fraction, left ventricle fractional shortening, left ventricular end-diastolic dimension and left ventricular end-systolic dimension of each group (*n* = 4). **E** Effect of STING knockdown on DOX-induced LDH, cTNT and CK-MB release (*n* = 4)

To confirm the effect of the cGAS-STING pathway on DOX induced cardiomyopathy, an AAV9-shRNA-STTING vector was used to induce STING knockdown mice. To induce STING knockdown in WT mice, the vector containing a green fluorescent protein (GFP) tag was administered intravenously 3 weeks prior to DOX treatment. The vector displayed a clear GFP signal in the hearts after 3 weeks of AAV9 infection, and western blot analysis verified shRNA-mediated STING expression repression in the hearts in contrasty with the control WT mice (Fig. 2A-E). Additionally, neither the control WT mice nor the STING knockdown animals differed in body weight or heart function. Subsequently, 14 days after DOX injection, we observed the role of STING in survival, cardiac injury and cardiac function among different groups, including PBS + WT group, PBS + STING knockdown group, DOX + WT group and DOX + STING knockdown group. As shown in Fig. 3A, the 14-day percent survival in PBS + WT group and PBS + STING knockdown group were nearly 100%, while DOX injection result in significantly decreased survival in DOX + WT group (50%, versus PBS + WT group, *P* < 0.05). And with the knockdown of STING in mice, the percent survival in DOX + STING knockdown group went up to 70% (versus DOX + WT group, *P* < 0.05). In addition, Plasm levels of LDH, cTnT and CK-MB activity were evaluated as myocardial damage markers to see if STING knockdown protected against DOX-triggered cardiac injury. Figure 3E illustrate that plasm LDH and CK-MB activities were remarkably higher in the DOX group in contrast with the PBS group, whereas STING knockdown may partially reverse this change.

At day 15, we used echocardiography to measure heart function in four groups. Treatment with doxorubicin reduced LV ejection fraction (LVEF) and fractional shortening (FS) in WT mice. End-systolic and end-diastolic volumes (LV dilation) were increased in Dox-treated WT mice. Above changes were partly improved in STING knockdown animals, as illustrated in Fig. 3C-D. Furthermore, doxorubicin-induced body weight loss and cardiac shrinkage were reversed when STING was knocked down (Fig. 3B).

### Knockdown of STING attenuated cardiac morphological alteration of DOX-treated mice

**Fig. 4:**
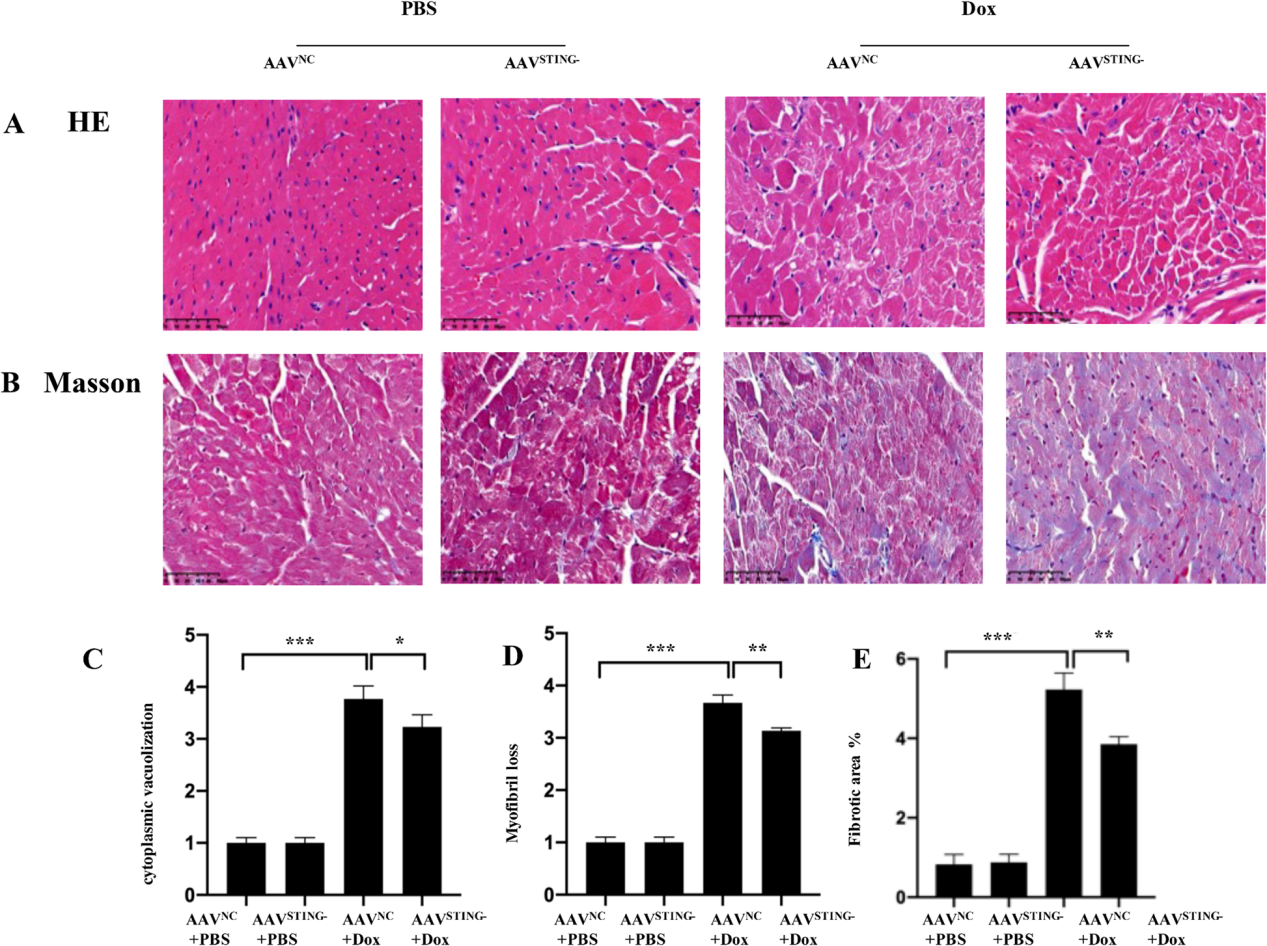
Light micrograph demonstrating the effect of STING knockdown on DOX-induced myocardial histological alterations. **A** Representative photomicrographs of mouse heart stained with H&E; **B** Representative photomicrographs of mouse heart stained with Masson;. Scale bar = 50 𝜇m. **C** Quantitative analysis for cytoplasmic vacuolization. **D** Quantitative analysis for myofibril loss. **E** Quantitative analysis for intestinal fibrosis (*n* = 4)

Previous studies have shown that Dox can cause a few structural changes to myocardium including cytoplasmic vacuolization, myofibril loss, and myocardial fibrosis [7, 8]. To demonstrate cardiac morphological alterations, sections of mouse heart tissue stained with H&E and Masson were examined by light microscopy. As shown in Fig. 4A, heart section from control showed normal cardiac morphology. Cytoplasmic vacuolization and myofibril loss was significantly (*p* < 0.05) increased in the Dox group, suggesting the heart has developed cardiac dysfunction. In comparison,

STING knockdown significantly reduced the vacuolization and myofibril loss (Fig. 4C-D).Further studies with Masson’s Trichrome staining revealed that DOX induced cardiac fibrosis compared with normal control while cardiac fibrosis with STING knockdown was much lower in cardiac tissue challenged with DOX (Fig. 4B). The quantitative analysis of cardiac fibrosis was demonstrated in Fig. 4E.

### Knockdown of STING suppressed cardiac inflammation, apoptosis of DOX-treated mice

**Fig. 5:**
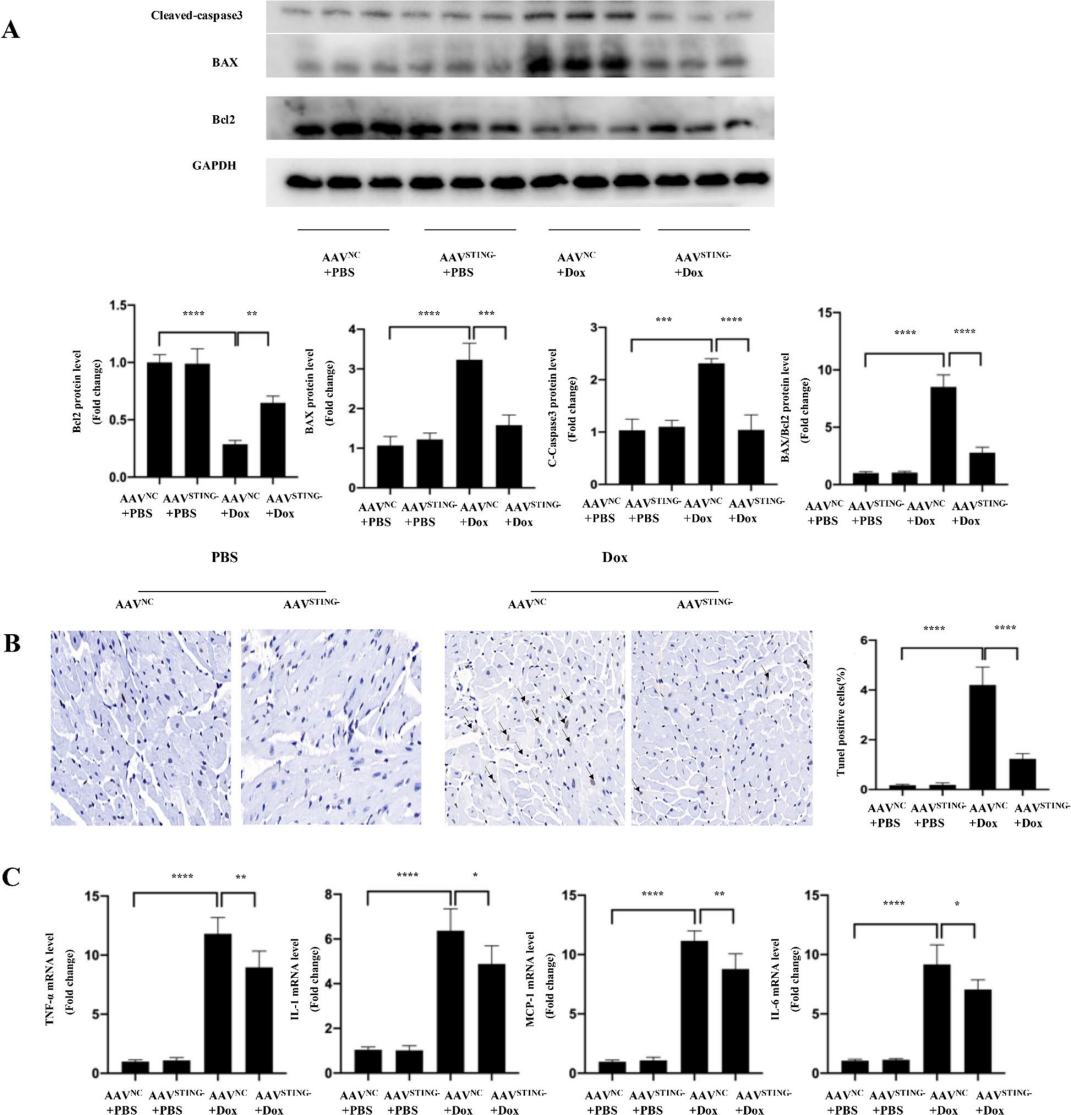
STING knockdown suppressed cardiac apoptosis inflammation, morphological alteration of DIC mice (**A**-**C**). **A**The protein levels of C-Caspase3, BAX and BCL-2 of each group after PBS or DOX injection. **B** TUNEL staining of each group after PBS or DOX injection (*n* = 4). The arrow indicated tunel positive cells. **C** mRNA analysis of inflammation-related genes of each group

Because doxorubicin’s toxic effects are mediated by inflammation and cardiac apoptosis, we investigated the effects of STING suppression on cardiac apoptosis, inflammation, and cell death. We employed western blot analysis to assess the apoptotic markers BAX and cleaved caspase-3, as well as the anti-apoptotic marker Bcl-2 (Fig. 5A). Meanwhile, we utilized Q-PCR to quantify the amounts of pro-inflammatory cytokines in cardiac tissue, such as p65, IL-1, IL-6, and TNF-α (Fig. 5C). In addition, we stained our tissue slices with TUNEL (Fig. 5B). STING suppression reduced apoptosis and inflammation in cardiomyocytes, as demonstrated by a lower ratio of C-Caspase3 to T-Caspase3, a lower ratio of BAX to BCL-2, and a lower number of TUNEL + cells, as well as lower levels of pro-inflammatory cytokines.

### cGAS-STING activation is involved in DOX-induced cell death in cultured cardiomyocytes

**Fig. 6:**
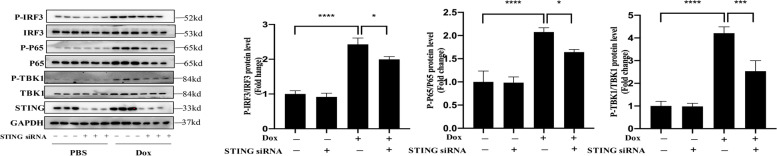
Suppressed DOX-induced phosphorylation of IRF3, TBK1 and p65 was observed by immunoblot analysis following STING knockdown in HL-1 cells following DOX treatment (1 μm for 24 h). Quantitative data represent the relative ratio to total IRF3, TBK1 or p65 (*n* = 6)

**Fig. 7:**
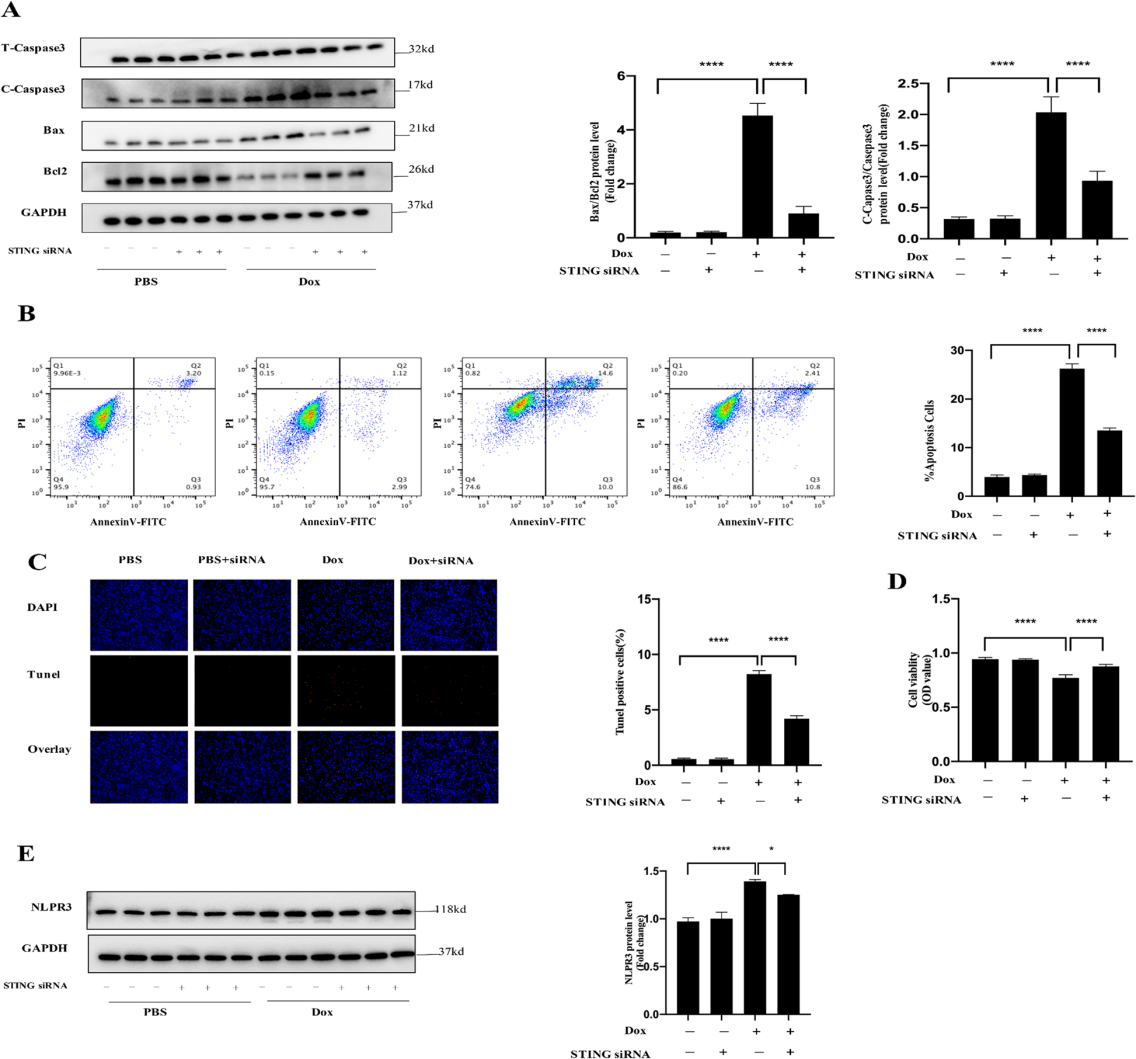
cGAS-STING activation is involved in DOX-induced cell death in cultured cardiomyocytes (**A**-**D**). **A **siRNA-mediated STING knockdown influenced the protein levels of C-Caspase3, Caspase3, BAX and BCL-2 in indicated groups in HL-1 cells (*n* = 6). **B**-**C** Flow cytometry assay and TUNEL staining showed that STING knockdown reduced cell apoptosis (*n* = 4). **D** CCK8 assay detection reveals that STING knockdown increased cell viability. **E **STING knockdown suppressed the protein levels of NLPR3

We investigated the influence of the cGAS-STING route in mouse HL-1 cardiomyocytes exposed to doxorubicin to elucidate the molecular mechanism by which the cGAS-STING casacade leads to cardiac injury in DOX-treated animals. The expression of cGAS and STING in HL-1 cardiomyocytes treated by doxorubicin (1µM, 24 h) was dramatically enhanced, which was consistent with the in vivo data. Furthermore, doxorubicin treatment increased phosphorylation of downstream molecules such as TBK1 IRF3 and NF-kB p65 (Fig. 6). Importantly, HL-1cells knocked down STING with siRNA revealed a reduction in dox-induced phosphorylation of STING downstream target proteins TBK1, IRF3, and NF-kB p65 (Fig. 6), resulting in reduced inflammation and apoptosis and higher cell survival (Fig. 7A-D).

Besides, we detected the protein expression level of NLPR3 in different groups. As expected, western blot shows NLRP3 infammasome was activated in Dox-treated HL-1cells, which was partly reversed by STING knockdown (Fig. 7E).

## Discussion

Doxorubicin’s therapeutic use is limited due to its cardiotoxicity, which eventually leads to irreversible cardiomyopathy and heart failure. The cGAS/STING cascade was shown to be active with doxorubicin treatment in this investigation. Furthermore, both in vivo and in vitro, STING knockdown may greatly reduce doxorubicin-induced cardiomyocyte damage. As a result, it is reasonable to assume that the cGAS/STING pathway maybe involved in the pathogenesis of DOX-induced cardiotoxicity Doxorubicin-Induced Cardiotoxicity and may represent a novel therapeutic target for DIC.

STING (also known as MPYS, MITA, TMEM173, and ERIS) is a protein found in the endoplasmic reticulum (ER) that transmits DNA-triggered signals and activates innate immunity [9, 10]. cGAS serves as a cytosolic sensor of DNA, mainly derived from pathogens. Activated cGAS triggers the generation of cGAMP, which binds to the STING in the ER [11]. STING then translocates from the ER to the perinuclear compartments, where it forms a complex with TANK-binding kinase 1 (TBK1), which is then delivered to the endolysosome, where TBK1 phosphorylates transcription factors such as IFN regulatory factor3 (IRF3) and nuclear factor NF-kB, triggering the expression of multiple inflammatory factors. Activation of the cGAS-STING signaling cascade abnormally has been linked to a variety of diseases [4]. Blocking cGAS-STING signaling in pressure overload-induced heart failure leads to lower levels of chemokines, inflammatory cytokines, and inflammatory cell infiltration in cardiac tissues, as well as better cardiac function [12]. Here, we employed an acute DIC model induced by single dose injection of doxorubicin to study a pathogenic role of STING-dependent cardiac damage. we demonstrated that in the onset of acute DIC, the mRNA and protein expression of cGAS-STING significantly increased 14 days of doxorubicin exposure, illustrating that cGAS-STING contributed to pathology of DIC. In addition, STING knockdown enhanced heart function and lifespan in mice exposed to doxorubicin. STING knockdown also reduced inflammation and apoptosis in mice following DOX injection or in cultured cardiomyocytes treated with DOX.

It has been shown in numerous research that activated STING accelerates the phosphorylation of protein kinases IκB kinase (IKK) along with TANK-binding kinase 1 (TBK1) [13, 14], triggering phosphorylation coupled with nuclear translocation of the transcription factor IRF3 [15]. P-IRF3 then dimerizes and translocates to the nucleus, where it regulates the production of inflammatory factors [16, 17]. Our findings are in line with those of these studies. The phosphorylation of TBK1 and IRF3 rose considerably after treatment with doxorubicin, and knocking down STING reduced these changes. As a result, the expression of a number of inflammatory factors was dramatically decreased.

NF-κB is a regulatory molecule that promotes the expression of some proinflammatory factors, and is also phosphorylated as a consequence of the full activation of TBK1 [18]. Many previous studies have indicated that activation of NF-κB play a significant role in the pathogenesis of Dox-induced cardiac damage [19, 20], including cardiomyocyte apoptosis [21]. In our study, we find that knockdown of STING contributes to diminish the phosphorylation and activation of p65 and thus attenuated cardiomyocyte apoptosis induced by doxorubicin treatment both in vivo and in vitro.

NLRP3 is a key mediator in the immune response’s initiation and inflammasome development. NLRP3 was shown to play a critical role in Dox-induced cardiotoxicity by affecting the release of pro-inflammatory cytokines and cardiomyocyte apoptosis or pyroptosis, according to growing research evidence [22–24]. Previous investigations have unveiled that the cGAS-STING is involved in inducing NLRP3 inflammasome [25, 26]. In our study, we also observed that silencing of STING contributes to attenuated the expression of NLRP3. Thus, we speculate that the protective effects of STING on Dox-induced cardiotoxicity is also partly associated with NLPR3.

**Fig. 8:**
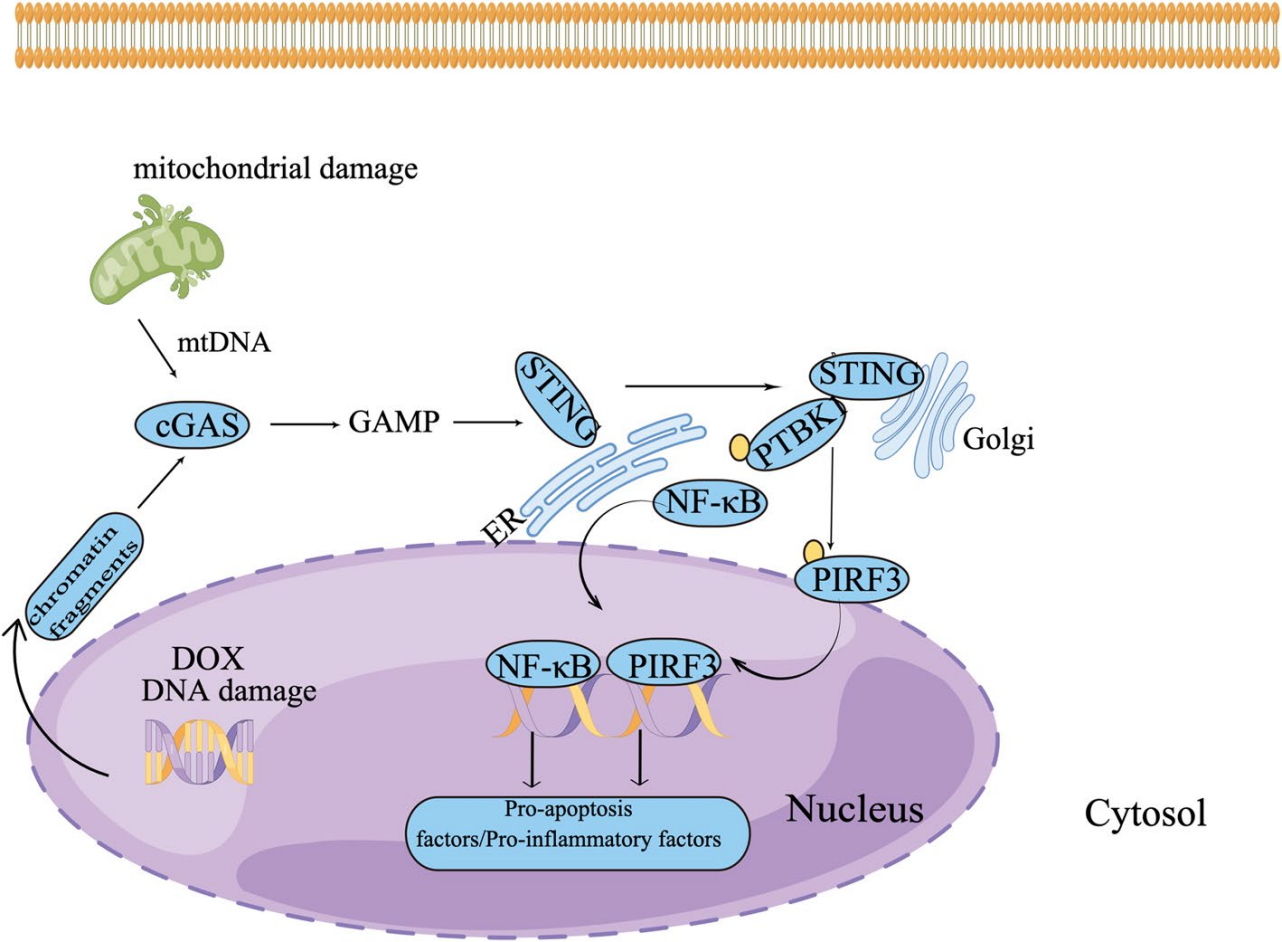
Schematic illustration of an impairment mechanism by cGAS-STING activation in DOX-induced cardiotoxicity

However, there are some limitations existing in this work. First, as acute DIC model is not the best model to mirror the chronic progressive nature of DIC, further studies are needed to confirm above findings in a chronic DIC model. Second, the AAV9 viral system that we used is that, despite higher cardio tropism, it also transduces other organs, and we cannot completely rule out effects mediated by transduction of other cell types or tissues. Third, the in vitro work is not confirmed in primary ventricular cardiomyocytes. Finally, further investigations are required to indentify whether STING knockdown will compromise the antitumor efficacy of DOX. In spite of these shortcomings, our findings disclosed that STING knockdown could attenuated DOX-triggered cardiac injury via dampening inflammation and apoptosis, suggesting that cGAS-STING pathway plays some role in Doxorubicin-Induced Cardiotoxicity. To our knowledge, this mechanism has been scarcely reported before, and the schematic diagram is illustrated in Fig. 8.

## Supplementary Information

**Additional file 1.** The raw WB data.

## Data Availability

All data supporting the fndings of this study are available within the published article.

## Abbreviations

AAV: Adeno-associated virus
CK-MB: Creatine kinase isoenzymes
DOX: Doxorubicin
DIC: DOX-induced cardiomyopathy
EF: Ejection fraction
FS: Fractional shortening
LDH: Lactate dehydrogenase
TUNEL: Terminal deoxynucleotidyl transferase-mediated dUTP nick end labeling
