# Supplementary material for: GAS-STING signaling plays an essential pathogenetic role in Doxorubicin-Induced Cardiotoxicity
Source: BMC Pharmacol Toxicol. 2023 Mar 24;24:19. doi: 10.1186/s40360-022-00631-0 (PMC10037834; doi:10.1186/s40360-022-00631-0)
Supplement: Supplementary file 1 — Additional file 1. The raw WB data. [file 40360_2022_631_MOESM1_ESM.docx]

## Supplementary Material

**The raw WB data**

**Fig1**

Fig1 GAPDH

Fig1 STING

Fig1 cGAS

**Fig2**

Fig2 STING

Fig2 GAPDH

**Fig4**

Fig4 GAPDH

Fig4 BCL2

Fig4 BAX

Fig4 CASPASE3

**Fig5**

Fig5 GAPDH

Fig5 IRF3

Fig5 PIRF3

Fig5 P65

Fig5 P-P65

Fig5 P-TBK1

Fig5 TBKI

Fig5 STING

**Fig6**

Fig6 GAPDH

Fig6 C-CASPASE3

Fig6 T-CASPASE3

Fig6 BCL2

Fig6 BAX

Fig6 GAPDH

Fig6 NLRP3
